# Routine Real-Time Cost-Effectiveness Monitoring of a Web-Based Depression Intervention: A Risk-Sharing Proposal

**DOI:** 10.2196/jmir.2592

**Published:** 2014-02-27

**Authors:** Klemen Naveršnik, Aleš Mrhar

**Affiliations:** ^1^Prototype AnalyticsSandoz Development CenterLek PharmaceuticalsLjubljanaSlovenia; ^2^Chair for Biopharmaceutics and PharmacokineticsFaculty of PharmacyUniversity of LjubljanaLjubljanaSlovenia

**Keywords:** depression, medical economics, value-based purchasing

## Abstract

**Background:**

A new health care technology must be cost-effective in order to be adopted. If evidence regarding cost-effectiveness is uncertain, then the decision maker faces two choices: (1) adopt the technology and run the risk that it is less effective in actual practice, or (2) reject the technology and risk that potential health is forgone. A new depression eHealth service was found to be cost-effective in a previously published study. The results, however, were unreliable because it was based on a pilot clinical trial. A conservative decision maker would normally require stronger evidence for the intervention to be implemented.

**Objective:**

Our objective was to evaluate how to facilitate service implementation by shifting the burden of risk due to uncertainty to the service provider and ensure that the intervention remains cost-effective during routine use.

**Methods:**

We propose a risk-sharing scheme, where the service cost depends on the actual effectiveness of the service in real-life setting. Routine efficacy data can be used as the input to the cost-effectiveness model, which employs a mapping function to translate a depression specific score into quality-adjusted life-years. The latter is the denominator in the cost-effectiveness ratio calculation, required by the health care decision maker. The output of the model is a “value graph”, showing intervention value as a function of its observed (future) efficacy, using the €30,000 per quality-adjusted life-year (QALY) threshold.

**Results:**

We found that the eHealth service should improve the patient’s outcome by at least 11.9 points on the Beck Depression Inventory scale in order for the cost-effectiveness ratio to remain below the €30,000/QALY threshold. The value of a single point improvement was found to be between €200 and €700, depending on depression severity at treatment start. Value of the eHealth service, based on the current efficacy estimates, is €1900, which is significantly above its estimated cost (€200).

**Conclusions:**

The eHealth depression service is particularly suited to routine monitoring, since data can be gathered through the Internet within the service communication channels. This enables real-time cost-effectiveness evaluation and allows a value-based price to be established. We propose a novel pricing scheme where the price is set to a point in the interval between cost and value, which provides an economic surplus to both the payer and the provider. Such a business model will assure that a portion of the surplus is retained by the payer and not completely appropriated by the private provider. If the eHealth service were to turn out less effective than originally anticipated, then the price would be lowered in order to achieve the cost-effectiveness threshold and this risk of financial loss would be borne by the provider.

## Introduction

Pricing and reimbursement decisions for new health care interventions are key to patient access to these treatments. Once an intervention is approved (based on evidence of its safety, efficacy, and quality), decision makers are faced with finding the value of the new treatment. Value statements are based on the objective of the health care system, which is normally maximization of health. Cost-effectiveness (CE) analysis is used to calculate the costs per unit of health (ie, quality-adjusted life-year [QALY]) for a given intervention. Generally, an intervention is considered cost-effective if its incremental cost-effectiveness ratio (ICER; unit: €/QALY) is below a predetermined threshold.

The level of CE threshold, adopted by the United Kingdom’s National Institute for Health and Care Excellence (NICE) lies in the range of £20,000-£30,000 per QALY [1]. A recent study reduced the estimate to £18,317 per QALY [2]. Interventions with an ICER below this range are generally deemed cost-effective. Interventions exceeding this range do not present good value for money and would generally not be implemented, unless there is significant value shown in other domains (such as low budget impact or a treatment for a priority disease area). The existence of such an explicit cost-effectiveness threshold provides a clear and predictable signal of value to the private sector (ie, drug manufacturers) because it specifies what decision makers will regard as being cost-effective [3]. A value-based approach enables companies to reduce their prices to levels that assure that their products are cost-effective. On the other hand, companies may respond to such a policy by raising their prices well above the production costs if the perceived value of the product is still high [4]. In either case, the decision maker’s goal is achieved, since health is purchased at a price below the cost-effectiveness threshold.

The reimbursement process has traditionally been binary: an intervention is either reimbursed or not. However, policies have emerged that expand the options, for example, linking coverage to evidence development [5]. Where data are insufficient to take an informed view on cost-effectiveness, then a risk-sharing approach could be adopted. This would require the company and payer to agree to a contract where the cost of the drug is reimbursed, contingent on claims of clinical effectiveness being realized in practice. If expected outcomes are not achieved, prices would need to be changed [6]. There is a surge of interest in risk-sharing schemes between health care payers and pharmaceutical companies in Europe. Performance-based risk sharing could produce efficient market equilibrium, achieved by adjustment of the price and post-launch evidence collection [7]. The effectiveness of the existing contracts, however, is limited, particularly due to high administration costs, lack of transparency, and conflicts of interest [8].

The intervention studied here is a novel eHealth service to support the treatment of patients with depressive symptoms, which aims at improving medication adherence and collaborative care management by combining Web-based and mobile-based systems. The eHealth service is applied in addition to the usual care over the Internet and mobile phones and allows active patient engagement and care management performed by trained psychologists and has been described previously [9]. Details on the intervention are included in Multimedia Appendix 1. The intervention was found to be very cost-effective in a previous study [10] with an ICER of €1430/QALY. There was, however, a high degree of uncertainty because efficacy data were based on a small pilot trial (46 patients) with high attrition. Varying assumptions regarding dropout pattern resulted in significantly lower cost-effectiveness [10]. Evidence regarding long-term benefits resulting from routine use is not available, and the risk that the same efficacy is not realized in real life would have to be borne by the payer if the treatment were to be reimbursed based on the existing data. Since social decision makers are not risk taking [11], we examine how this risk could be shared with (or shifted to) the service provider.

The purpose of our paper is to identify if and how an efficient risk-sharing scheme for a new depression treatment can be implemented. The objective of the agreement is to guarantee that the payer purchases health at a cost below the CE threshold. Our aim is to provide a clear definition of (1) what data need to be collected during routine use as well as how these data are used to calculate real-time cost-effectiveness, and (2) how the price is adjusted in order to meet the CE threshold.

## Methods

### Design

We propose a performance-based risk-sharing agreement to provide coverage “only in research” [12]. This means that the intervention is reimbursed only on condition that efficacy data are gathered for every patient during routine use. The objective is to periodically recalculate cost-effectiveness of the service based on the obtained data and adjust its price accordingly. If the recalculated ICER exceeds the CE threshold, the price is to be reduced. On the other hand, if the recalculated ICER is below threshold, the price may be increased and the surplus shared between the payer and provider.

### Evidence to be Collected During Routine Use

**Figure 1:**
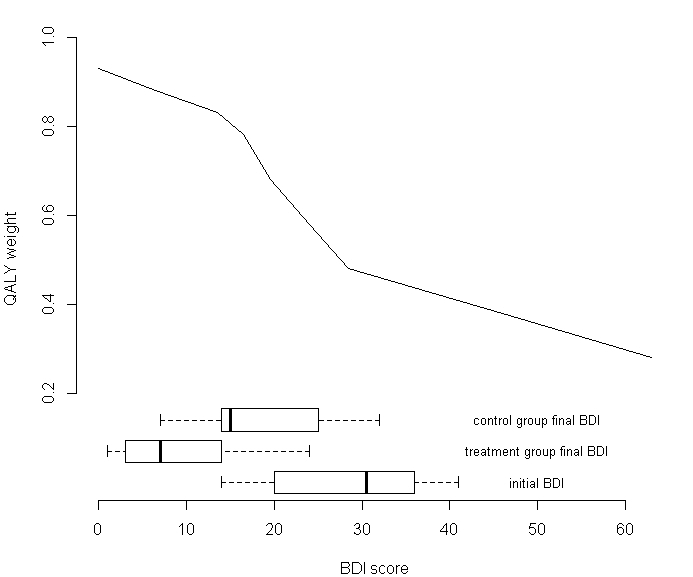
Box-plots of BDI scores for patients, entering the pilot trial (initial BDI) and treatment/control groups at the end of the pilot trial (piecewise linear line shows how BDI score [horizontal axis] is mapped onto the QALY scale [vertical axis]).

Intervention efficacy is currently the main source of uncertainty. Long-term improvement of quality of life should thus be investigated during routine use. We propose that decline of depression severity be monitored by the Beck Depression Inventory (BDI-II) scale [13], self-administered by the patients. As evaluated in the pilot trial [9], the BDI score is collected at enrollment and at regular intervals during use. The questionnaire can be applied via the Health service platform and requires very few additional resources. Current mean estimates of BDI improvement, based on the pilot trial are shown as box-plots in Figure 1. The average BDI score for patients entering the trial was 29 (“initial BDI”). Half a year later, the score was reduced to 18 and 10 for the control and treatment arms respectively.

A previously published mapping function [10] is then used to convert BDI score into quality of life weight (Figure 1). Mapping of BDI scores onto the QALY scale is encouraged, since generic instruments such as EQ-5D lack sensitivity in measuring quality of life in mental disorders [14]. Collecting both EQ-5D and BDI data would be useful in order to validate the existing mapping function. Mapping BDI data onto QALY in the pilot trial (Figure 1) resulted in a mean initial QALY weight 0.53, which increased to final values 0.72 and 0.83 for the control and treatment arm respectively.

## Results

### Price Recalculation

**Figure 2:**
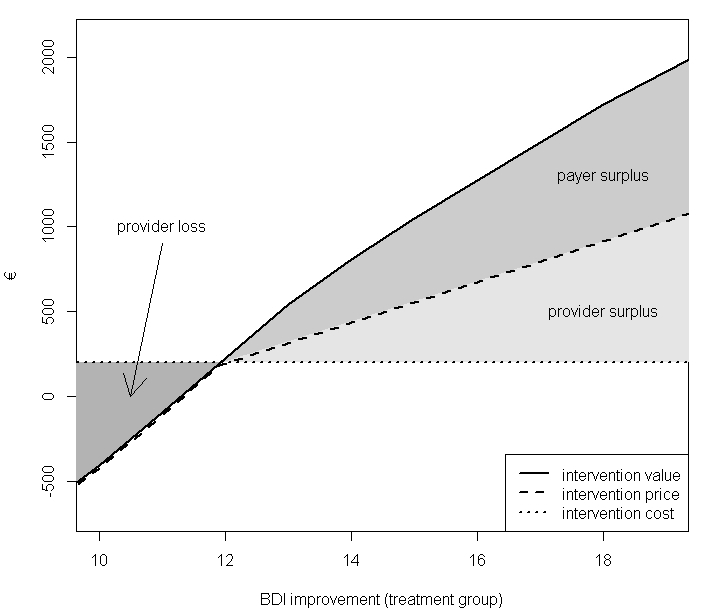
Relation between intervention value, price, and cost, depending on the actual efficacy (BDI improvement) during routine use (intervention value calculated using €30,000/QALY threshold; shaded regions depict provider loss and social surplus, shared between the provider and payer at an example ratio of 1:1 [50% private appropriation level, intervention cost at €200 per patient]).

How and when the evidence will be used to adjust the price should be clearly stated in the agreement. A previously published cost-effectiveness model [10] is used in order to calculate intervention value based on the data gathered along routine use. The economic model is detailed in Multimedia Appendix 1. Patients’ BDI score improvement dictates the amount of QALYs gained (through the mapping function in Figure 1). A cost-effectiveness threshold of €30,000/QALY is used as an example in our calculations but would be adapted according to the payers’ threshold. Explicitly stating the threshold monetary value for a QALY in turn allows calculation of the value of intervention. The price paid by the health authority should reflect the intervention’s value, regardless of the cost borne by the provider (which is estimated at €200 per patient). Figure 2 shows how the price would be set, depending on the actual efficacy (BDI depression score reduction). The plot has two distinct regions, separated by the threshold BDI improvement level (11.9), which correspond to an ICER of €30,000/QALY (using the pilot trial BDI data, where control group BDI score improved by 10.9 points on average). In the low efficacy range (<11.9 BDI points), the provider is expected to reduce the price so that it does not exceed the intervention’s value. Such a scenario would represent financial loss to the provider, since the price would have to be set below the actual intervention cost. On the other hand, if it turned out that efficacy is higher (>11.9 BDI points), intervention value would exceed its cost. The price could be raised in this case to a level not exceeding intervention value. Such a model would allow the total economic surplus (value minus cost) to be shared between the payer and provider. A portion of the surplus would go to the payer, since the intervention’s value would exceed its price. The provider, on the other hand would retain the difference between the price charged and the intervention cost as profit. The actual service efficacy found in the pilot trial was a 19-point improvement on the BDI scale for the treatment arm. This translates into a value of €1900 (per patient per year), which is significantly above the estimated service cost.

The current market level of private appropriation in health technologies was estimated at about 15% [4]. An appropriate ratio of payer versus provider surplus would have to be agreed upon and would depend for instance on the perceived risk of provider loss and the amount of research and development investment in the creation of the service. Such an agreement would also prevent the service provider from gradually increasing the price until the cost-effectiveness is at (or just below) the threshold level.

### Data Collection

The key issue to be addressed is how to ensure a transparent mode of data collection. Practicality, small administrative burden, and low costs dictate that data be gathered through the provider’s IT system. Means of ensuring data integrity would have to be set in place, as there is a clear conflict of interest if the provider is also the data collector. The data generation scheme would also need to provide control group data, since there is only a small historical control group available so far. This calls for a randomized design where an appropriate target control group size would need to be prespecified.

An important issue in analyzing the pilot trial data was how to handle a large body of missing data. Dropout rates up to 60% were observed [9] and should be reduced during routine use to reach a prespecified minimum response rate. Measures to reduce missing data could include a run-in period, limiting participants’ burden in the data collection stage, and collecting data also for dropouts [15]. A major methodological issue to be addressed is how to handle missing data in the statistical analysis. The use of available cases for four alternative data imputation scenarios in the analysis of pilot trial data resulted in markedly different estimates of ICER. Handling missing BDI data should clearly be specified when calculating price adjustment, as well as a sensitivity analysis performed with alternative methods to check the robustness of results [16].

## Discussion

### Principal Findings

New drugs could have a substantial beneficial impact on service delivery and patient safety in practice, but it has been difficult to prove this within the confines of a phase III trial [8]. The situation is similar for a novel eHealth depression service, which appears to be very cost-effective yet has considerable uncertainty, since efficacy data are available only from a single pilot clinical trial. The decision maker must now consider whether the benefits of immediate access to the technology exceed the potential risk of the service being cost-ineffective in real practice. If the service is not implemented, a considerable amount of health may be forgone. On the other hand, if the service is implemented and turns out to be less cost-effective in actual practice, money invested could have been better spent on other health care interventions.

Due to its nature (applied over the Internet and mobile technologies), the eHealth depression service lends itself to routine data collection. In fact, many eHealth and mHealth interventions allow collection of effectiveness data during routine use, particularly if effectiveness can be self-reported by the patient, for instance by questionnaires. Such data can be very useful for an economic or any other evaluation, as it does not suffer from sampling limitations (ie, it effectively samples the whole treated population). This opens an array of options for market entry, such as risk-sharing agreements.

We propose that effectiveness data be gathered throughout routine use, once the service is launched. These data points, in turn, can be used to iteratively reassess the intervention’s cost-effectiveness. This allows a risk averse payer to be charged with a price no higher than the value it represents. There has been a lot of skepticism regarding risk-sharing agreements, propelled by the UK multiple sclerosis patient access scheme, which was perceived as a “costly failure” [17]. We have thus addressed components of a potential risk-sharing agreement, most likely to be critical to its effectiveness. Three key aspects were how to ensure transparency of data collection and evaluation, handling of missing data, and obtaining control group data.

The core component of the proposed scheme is price recalculation based on the observed effectiveness. This is where risk is shared between the provider (price goes down if effectiveness is low) and the payer (price increases if the observed value is high). The price ceiling is set with regards to the explicit cost-effectiveness threshold, set by the decision maker. This ensures that the service represents good value to the payer. Furthermore, we propose a novel business model of surplus sharing between the payer and the service provider. An appropriate provider/payer surplus ratio ensures that a portion of the profits due to high cost-effectiveness is appropriated by the payer (health insurance) and that the service provider still retains an incentive to optimize the service in order to achieve high effectiveness.

### Limitations

**Figure 3:**
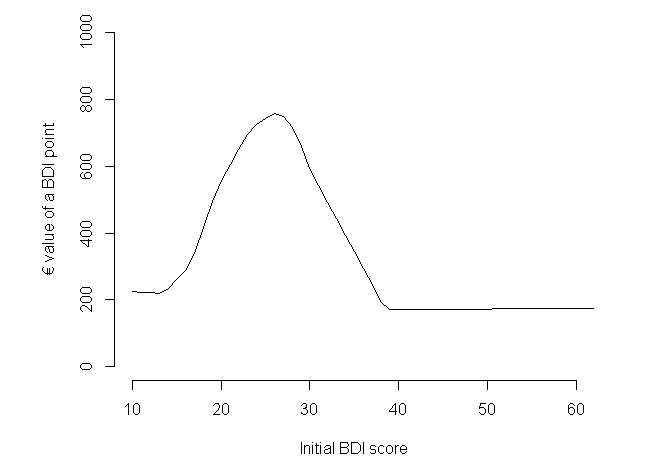
Value of a BDI point improvement (treatment minus control) as a function of the patient's initial BDI score (calculation based on a treatment effect of 10 BDI points).

The service provider would benefit from patient selection based on their initial BDI score. The value of a BDI point is not uniform across the whole BDI range (Figure 3) due to varying slope of the mapping function (Figure 1). This could give the provider an incentive to include patients only with initial BDI scores at around 25, since this would maximize treatment effect and value.

Another shortcoming is source of cost data used in the health economic model. Costs due to depression states were taken from a similar intervention in the United Kingdom, and these may be different in other settings. Although ICER was found to be insensitive to depression-related costs [10], these cannot be ignored, particularly because using the service could encourage patients to consume more health care resources (specialist visits, drugs, etc). Depression costs are significant in the indirect domain (productivity loss from absenteeism and presenteeism). These costs are not included in the model and would likely be reduced by treating depression. A conservative approach would thus assume that any potential cost increase due to higher health care consumption due to service would be offset by a reduction in indirect costs due to health improvement.

Voluntary consent to research, free of coercion, or penalty for refusal is a basic requirement for research involving competent adults [18]. When a treatment is offered in research only, this may have ethical implications [19]. If a patient was to decline reporting BDI data, would they still be eligible to use the eHealth service? If not, that may result in patient coercion to participate in the study. Random allocation to control and treatment groups could be an ethical issue if the service became standard treatment, since the service would be denied to patients, randomized to the control group. They would still receive treatment as usual but would be denied a potentially effective eHealth service. If this was deemed unacceptable, then a randomized control group would not be available. An alternative control group strategy is to follow up on patients that chose not to use the service (for instance due to lack of Internet access) and thus receive treatment as usual.

Our analysis is based on the notion that health (as measured by quality-adjusted life-years) is the only domain representing value to the payer. It should be noted that, depending on the perspective of the decision maker, other domains should also be taken into account, including severity of illness, unmet medical need, and wider societal considerations such as impacts on caregivers and equality [20]. Since value in other domains is difficult to monetize, we excluded them from the analysis.

Service cost is one of the variables in the price recalculation model. The current rough estimate of intervention cost is €200 per patient. The actual intervention cost (per patient) during routine use is likely to depend on the total number of patients enrolled in the program. Since cost is known only to the service provider, this could be exploited in order to achieve a higher price.

### Conclusion

We have shown how to shift the risk due to uncertainty from the health care payer to the service provider. Such an agreement requires a value-based pricing scheme and continuous efficacy monitoring throughout routine use. The latter is easily implemented in eHealth or mHealth interventions as long as a patient-reported measure of health is available. We proposed a flexible pricing scheme that allows economic surplus to be shared between the payer and provider in case of high treatment efficacy during routine use and allows the payer to avoid any potential loss in case of low efficacy.

## Multimedia Appendix 1

Intervention pilot study details.

## Abbreviations

BDI: Beck Depression Inventory
CE: cost-effectiveness
ICER: incremental cost-effectiveness ratio
QALY: quality-adjusted life-years

## References

[1] (2008). NICE.

[2] Claxton K, Martin S, Soares M, Rice N, Spackman E, Hinde S, Devlin N, Smith PCSculpher M (2013). CHE Research Paper 81.

[3] Claxton K (2007). OFT, VBP: QED?. Health Econ.

[4] Jena AB, Philipson TJ (2008). Cost-effectiveness analysis and innovation. J Health Econ.

[5] Walker S, Sculpher M, Claxton K, Palmer S (2012). Coverage with evidence development, only in research, risk sharing, or patient access scheme? A framework for coverage decisions. Value Health.

[6] Office of Fair Trading (2007). OFT: The Pharmaceutical Price Regulation Scheme: an OFT market study.

[7] Towse A, Garrison LP (2010). Can't get no satisfaction? Will pay for performance help?: toward an economic framework for understanding performance-based risk-sharing agreements for innovative medical products. Pharmacoeconomics.

[8] Adamski J, Godman B, Ofierska-Sujkowska G, Osińska B, Herholz H, Wendykowska K, Laius O, Jan S, Sermet C, Zara C, Kalaba M, Gustafsson R, Garuolienè K, Haycox A, Garattini S, Gustafsson LL (2010). Risk sharing arrangements for pharmaceuticals: potential considerations and recommendations for European payers. BMC Health Serv Res.

[9] Meglic M, Furlan M, Kuzmanic M, Kozel D, Baraga D, Kuhar I, Kosir B, Iljaz R, Novak Sarotar B, Dernovsek MZ, Marusic A, Eysenbach G, Brodnik A (2010). Feasibility of an eHealth service to support collaborative depression care: results of a pilot study. J Med Internet Res.

[10] Naveršnik K, Mrhar A (2013). Cost-effectiveness of a novel e-health depression service. Telemed J E Health.

[11] Zivin JG (2001). Cost-effectiveness analysis with risk aversion. Health Econ.

[12] Trueman P, Grainger DL, Downs KE (2010). Coverage with Evidence Development: applications and issues. Int J Technol Assess Health Care.

[13] Beck AT, Steer RA, Brown G (1996). BDI-II, Beck depression inventory: manual.

[14] Brazier J (2010). Is the EQ-5D fit for purpose in mental health?. Br J Psychiatry.

[15] Li T, Hutfless S, Dickerson K, Scharftstein D, Neaton J, Hogan J, Little R, Daniels M, Roy J, Mor V, Law A (2012). Minimal Standards in the Prevention and Handling of Missing Data in Observational and Experimental Patient Centered Outcomes Research.

[16] Marshall A, Billingham LJ, Bryan S (2009). Can we afford to ignore missing data in cost-effectiveness analyses?. Eur J Health Econ.

[17] Raftery J (2010). Multiple sclerosis risk sharing scheme: a costly failure. BMJ.

[18] Emanuel EJ, Wendler D, Grady C (2000). What makes clinical research ethical?. JAMA.

[19] Miller FG, Pearson SD (2008). Coverage with evidence development: ethical issues and policy implications. Med Care.

[20] Sussex J, Towse A, Devlin N (2013). Operationalizing value-based pricing of medicines : a taxonomy of approaches. Pharmacoeconomics.

