# Supplementary material for: Routine Real-Time Cost-Effectiveness Monitoring of a Web-Based Depression Intervention: A Risk-Sharing Proposal
Source: J Med Internet Res. 2014 Feb 27;16(2):e67. doi: 10.2196/jmir.2592 (PMC3961743; doi:10.2196/jmir.2592)
Supplement: Supplementary file 1 [file jmir_v16i2e67_app1.pdf]

Multimedia Appendix 1. Intervention pilot study details

Intervention: Improvehealth.eu service was applied in addition to the usual care over the internet and mobile phones. It consisted of (i) a Web-based information and communication technology system, designed to support collaborative care management and active patient engagement, and (ii) online and phone-based care management performed by trained psychologists. The control group received treatment as usual - that is physician visits and antidepressant treatment. The eHealth service resulted in higher medication adherence and better outcomes, relative to the usual care. The main outcome measure was Beck Depression Inventory (BDI-II) score, which is a 21-item self-report scale used to determine depression severity. Items are scored on a 0–3 scale giving a total range of 0–63. Treatment as usual improved BDI-II score for 11 points and the eHealth intervention nearly doubled this effect (20 point improvement on the BDI-II score). Inclusion criteria were: a diagnosis of depression or mixed anxiety and depression disorder for the first time or after a remission of at least 6 months; introduction of antidepressant treatment in the last 10 days; regular use of Internet and mobile phone and a BDI-II score of 14 or more.

Cost effectiveness model

Model: The structure of our model followed the pilot efficacy trial. Patient were enlisted to either arm (treatment as usual or eHealth intervention) and followed for 6 months. The patient's BDI-II score was assumed to have linearly changed from the initial value to the final measured value after the 6-month follow-up. After that, it was assumed that the BDI-II score returned to the initial value linearly through the next 6-month period. The primary efficacy measure in the pilot was BDI-II and was transformed into utility weights (QALY) by applying a mapping function (figure 1): BDI-II cutoff points for severe, moderate and mild depression were 28.5, 19.5 and 13.5, respectively and utility values were taken from the literature (values of 0.38, 0.58 and 0.78, respectively). These utility values were assigned to the mean BDI-II score of each depression state and a piecewise linear function fitted through the BDI-II scores at the middle and borders of each severity state.

Costs were modeled in two ways. One source of costs is the intervention itself (estimated at €200) and the other comes from the use of other services. Healthcare costs (other than eHealth service costs) for four depression severity states were obtained from the literature did not include productivity or lost employment costs.

The model was evaluated by bootstrapping patient BDI-II scores from the pilot trial and calculating cost per QALY gained.

Value based pricing

Intervention value as a function of efficacy (figure 2) was evaluated according the net health benefits approach (Stinnett AA, Mullahy J: Net health benefits: a new framework for the analysis of uncertainty in cost-effectiveness analysis. Med Decis Making. 1998 Apr-Jun;18(2 Suppl):S68-80). Value was expressed as:

Net health benefit = incremental QALY * 30000€/QALY – incremental cost (€).

Incremental costs and QALYs were calculated for various BDI-II score improvement levels using the original cost-effectiveness model and adjusting the 6-month treatment group BDI values as the independent variable.
